# ﻿A new species of *Homatula* (Teleostei, Cobitoidea, Nemacheilidae) from the Pearl River drainage, Yunnan, China

**DOI:** 10.3897/zookeys.1089.77203

**Published:** 2022-03-17

**Authors:** Rui Min, Yahui Zhao, Jingsong Shi, Junxing Yang

**Affiliations:** 1 Kunming Natural History Museum of Zoology, Kunming Institute of Zoology, Chinese Academy of Sciences, Kunming, Yunnan 650223, China; 2 Key Laboratory of Zoological Systematics and Evolution, Institute of Zoology, Chinese Academy of Sciences, Beijing 100101, China; 3 Key Laboratory of Vertebrate Evolution and Human Origins of Chinese Academy of Sciences, Institute of Vertebrate Paleontology and Paleoanthropology, Chinese Academy of Sciences, Beijing 100044, China; 4 State Key Laboratory of Genetic Resources and Evolution, Kunming Institute of Zoology, The Innovative Academy of Seed Design, Chinese Academy of Sciences, Kunming, Yunnan 650223, China; 5 Yunnan Key Laboratory of Plateau Fish Breeding, Kunming Institute of Zoology, Chinese Academy of Sciences, Kunming, Yunnan 650223, China

**Keywords:** Molecular phylogeny, morphology, Nanpanjiang River, osteology, taxonomy

## Abstract

Based on morphological and molecular analysis of *Homatula* species distributed in the Nanpanjiang River in Yunnan, China, we described a new species, *Homatularobusta***sp. nov.** It differs from its congeners by a combination of the following characters: naked and robust body with well-developed crests (caudal peduncle depth as a percentage of its length: 70.5–78.5%); lateral line complete; median notch on lower jaw; median gap on lower lip; three pairs of short barbels, with maxillary barbels extending posteriorly to anterior edge of eyes; branched dorsal-fin rays 8½; and vertebrae 37–39. It can further be distinguished from *H.nanpanjiangensis* by several differences of the caudal skeleton such as the number of hypural elements, the presence of epurale and the shape of neural and haemal spines. Phylogenetic analysis of the mitochondrial cytochrome c oxidase subunit I (COI) gene indicated that the new species represents an independent lineage. It is separated from other *Homatula* species by a minimum of 5.3% Kimura-2-parameter distance in the COI gene. Furthermore, we confirmed that *Homatulawenshanensis* should be a member of *Homatula* based on both skeleton and molecular evidence.

## ﻿Introduction

*Homatula*, a group of benthic nemacheilids distributed in the eastern slope of the Qinghai-Tibetan Plateau, was established by Nichols in 1925 based on *Nemachiluspotanini* Günther, 1896 from the Minjiang River (a tributary of the Yangtze River, Sichuan, China) (Kottelat 1990; Bănărescu and Nalbant 1995; Min et al. 2012). Species of *Homatula* are characterized by the crests along the dorsal and ventral margins of the caudal peduncle supported by rudimentary procurrent caudal-fin rays, the presence of a degenerated non-ossified secondary gas-bladder chamber, and a medium to large-sized body with a maximum standard length of 190 mm (Zhu 1989; Kottelat 1990; Bănărescu and Nalbant 1995; Endruweit et al. 2018).

Currently, 21 valid species are recognized, mostly distributed in China, and only one species is recently reported from Vietnam (Nguyen et al. 2021; Zhou et al. 2021). In China, six species are in the Palaearctic drainages of the Yangtze and Yellow River, four in the Lancangjiang River (upper reaches of the Mekong River), three in the Nujiang River (Salween River), four in the Red River, and three in the Nanpanjiang River (upper reaches of the Pearl River), respectively. None of them is distributed across these large river systems.

Three *Homatula* species have been reported from the Nanpanjiang River, i.e., *H.oligolepis* (Cao & Zhu, 1989), *H.longidorsalis* (Yang, Chen & Kottelat, 1994) and *H.nanpanjiangensis* (Min, Chen & Yang, 2010). In 2009, a medium-sized loach was collected from Luoping County, Yunnan Province, China, which belongs to the Nanpanjiang River drainage. By comparing it to other *Homatula* species, especially the species distributed in the Nanpanjiang River, we describe it as a new species here.

## ﻿Materials and methods

All specimens were collected in 2009 from Yunnan Province, China and they were fixed either in 95% ethanol or 10% formalin and transferred to 75% ethanol for preservation. For DNA analysis, tissue samples from the left pelvic fin were excised from one or more specimens and placed in 95% ethanol. General methods for measurements and counts were done following Kottelat (1990), pore counts followed Armbruster (2012). Measurements were made with digital calipers to the nearest 0.1 mm from the left side. X-ray images were used to count vertebrae and simple fin rays. Lateral line pores and rays of paired fins were counted under a binocular microscope. The Weberian apparatus was counted as four vertebrae. Caudal vertebrae encompassed all centra bearing a haemal spine, including the urostyle, which was counted as one vertebra. Eye diameter was measured horizontally. Body depth was measured at the dorsal-fin origin. Lateral head length was measured from snout tip to the posterior margin of the operculum, excluding the opercular membrane. Examined specimens were deposited in the collection of the Kunming Natural History Museum of Zoology, Kunming Institute of Zoology (KIZ), Chinese Academy of Sciences.

In order to compare skeletal morphology, we applied Computed Microtomographic (μCT) scans of the holotype of *H.robusta* (KIZ 2009000125), a paratype of *H.nanpanjiangensis* (KIZ 1994000029) and a specimen of *H.wenshanensis* (KIZ 2014005686). Specimens were scanned using a GE Phoenix v|tome|x m dual tube 300/180 kV system in the Key Laboratory of Vertebrate Evolution and Human Origins, Institute of Vertebrate Paleontology and Paleoanthropology (IVPP), Chinese Academy of Sciences. The specimen was scanned with an energy beam of 80 kV and a flux of 80*μA using a 360° rotation and then reconstructed into a 4096*4096 matrix of 1536 slices. The final CT reconstructed skeleton images were exported with a minimum resolution of 6.099 μm. The skeleton images were exported from the virtual 3D model which was reconstructed using Volume Graphics Studio 3.0. Osteological terminology generally follows that of Prokofiev (2009) and Conway (2011) with modifications.

DNA was extracted from fin tissues using standard phenol-chloroform extraction (Sambrook et al. 1989). Mitochondrial cytochrome c oxidase subunit 1 (COI) was amplified by polymerase chain reaction (PCR). The PCR protocols were conducted in 50-µl reactions as follows: initial denaturation step at 95 °C for 5 min, 35 cycles at 94 °C for 30 s, 56 °C for 45 s, and 72 °C for 1 min, and final extension at 72 °C for 10 min. The primers used for COI were LCOIa (CCT ACC TgT ggC AAT CAC RCg C), HCOI (gTg AAT Agg ggg AAT CAg Tg) (Liu et al. 2012). Fragments were sequenced by the Shanghai DNA Biotechnologies Company (China). DNA sequences were aligned using default settings in MAFFT v7 (http://mafft.cbrc.jp/alignment/server/) (Katoh and Standley 2013), and, if necessary, adjusted by eye. MEGA7 (Kumar et al. 2016) was used to calculate the Kimura’s 2-parameter genetic distance (K2P). The phylogeny was analyzed using MrBayes 3.2 (Ronquist et al. 2012) with the generalized time reversible model (nst = 6) and the gamma-distributed rate variation and proportion of invariable positions (GTR+ I) for the COI datasets. We ran four simultaneous Monte Carlo Markov chains for 2 000 000 generations, with sampling every 1000 generations, and the first 25% of samples were discarded as burn-in.

### ﻿Comparative materials

*Homatulalongidorsalis* (Yang, Chen & Kottelat, 1994) (*N* = 24): Holotype: China; Yunnan, Yiliang, Jiuxiang; KIZ 1987003989, 82.0 mm SL. Paratypes: China; Yunnan, Yiliang, Jiuxiang; KIZ 1987003990, 3991–3993, 5090, 5091, 5736–5752, 46.0–89.5 mm SL.

*Homatulananpanjiangensis* (Min, Chen & Yang, 2010) (*N* = 20): Holotype: China; Yunnan, Qujing, Luoping; KIZ 1994000023, 86.8 mm SL. Paratypes: China: Yunnan: Qujing: Luoping; KIZ 1994000018–22, 024–037, 72.4–89.7 mm SL.

*Homatulaoligolepis* (Cao & Zhu, 1989) (*N* = 2): China; Yunnan, Zhanyi; KIZ 1985000829, KIZ 652099, 138.2–170.7 mm SL.

*Homatulapotanini* (Günther, 1896) (*N* = 5): China; Sichuan, Meishan; KIZ 2010000266; China; Sichuan, Luoshan; KIZ 2010000279–82, 70.6–80.1 mm SL.

*Homatulavariegata* (Dabry de Thiersant, 1874) (*N* = 9): China; Sichuan, Panzhihua; KIZ 2009002724–2727, 77.4–95.2 mm SL; China; Yunnan, Zhaotong, Yanjin; KIZ 2004008050, 52–53, 57, 61, 76.3–101.6 mm SL.

*Homatulalaxiclathra* Gu & Zhang, 2012 (*N* = 2): China; Shanxi: Ankang: Ningshan: Weihe River: KIZ 2012002359–60, 100.5–120.5 mm SL.

*Homatulaguanheensis* Zhou, Ma, Wang, Tang, Meng & Nie, 2021 (*N* = 6): China: Shanxi, Ankang, Ningshan, Yangtze River; KIZ 2005014508–13, 104.5–135 mm SL.

*Homatulawuliangensis* Min, Yang & Chen, 2012 (*N* = 34): Holotype: China; Yunnan, Jingdong; KIZ 2008008158, 181.9 mm SL. Paratypes: China; Yunnan, Jingdong; KIZ 2008008156–157, 159–172, 175–176, 179, 184, 197, 199–201, 203, 205, 207, 211, 214–215, 316–318, 64.6–191.1 mm SL.

*Homatuladisparizona* Min, Yang & Chen, 2013 (*N* = 21): Holotype: China; Yunnan, Wenshan, Xichou; KIZ 2012000623. Paratypes: China; Yunnan, Wenshan, Xichou; KIZ 2012000622, 624–634. China; Yunnan, Wenshan, Xichou; KIZ 2014005623–30, 62.8–92.4 mm SL.

*Homatulaacuticephala* (Zhou & He, 1993) (*N* = 26): China; Yunnan, Dali, Haixihai; KIZ 2008005990–6015, 33.7–51.5 mm SL.

*Homatulaanguillioides* (Zhu & Wang, 1985) (*N* = 12): China; Yunnan, Dali, Eryuan, KIZ 2008006532–6543, 68.8–143.3 mm SL.

*Homatulapycnolepis* Hu & Zhang, 2010 (*N* = 6): China; Yunnan, Dali, Yangbi; KIZ 1998004817, 19, 22, 25, KIZ 2009005288, KIZ 2009005388, 120.1–177.1 mm SL.

*Homatulachange* Endruweit, 2015 (*N* = 12): Holotype: China; Yunnan, Puer, Jiangcheng; KIZ 2012004205, 107.6 mm SL. Paratypes: China; Yunnan, Puer, Jiangcheng; KIZ 2012004208, 4209, 4211, 4215–18, 4221–24, 37.9–76.5 mm SL.

*Homatulacoccinocola* Endruweit, Min & Yang, 2018 (*N* = 5): Holotype: China, Yunnan, Honghe; KIZ 2011002847, 99.6 mm SL. Paratypes: China, Yunnan, Honghe; KIZ 2012001866–1869, 51.1–79.0 mm SL.

*Homatulacryptoclathrata* Li, Che & Zhou, 2019 (*N* = 2): China; Yunnan, Lincang; KIZ 2005012637, 39, 91.3–100 mm SL.

*Homatulawenshanensis* Li, Yang, Li & Liu, 2017 (*N* = 3): China; Yunnan, Wenshan; KIZ 2014005685–87, 60.2–110.9 mm SL.

We obtained information on *H.wujiangensis* Ding & Deng, 1990 from Ding and Deng (1990), on *H.anteridorsalis* Li, Che & Zhou, 2019 and *H.nigra* Li, Che & Zhou, 2019 from Li, Che and Zhou (2019), and *H.dotui* Nguyen, Wu, Cao & Zhang, 2021 from Nguyen et al. (2021).

GenBank Accession numbers are listed in Table 1.

**Table 1.** Voucher and Genbank numbers for study samples; sequences downloaded from GenBank are without voucher numbers.

Taxon	Voucher number	GenBank number
*Triplophysabrevicauda*	KIZ 050422024	MZ677092
*Triplophysascleroptera*	KIZ 20100076	MZ677093
*Triplophysaobscura*	–	MG238209
*Claeadabryi*	KIZ 2009003600	MZ677094
*Schisturafasciolata*	KIZ 2012003668	MZ677096
*Schisturamacrocephalus*	KIZ 2010003135	MZ677098
*Schisturalatifasciata*	KIZ CXY2008062	MZ677099
*Schisturacallichroma*	KIZ 200401055	MZ677095
*Schisturacaudofurca*	KIZ 20150307022	MZ677097
*Homatulawujiangensis*	–	IHB0301075
*Homatulapotanini*	KIZ 2010000235	MZ677100
*Homatulapotanini*	KIZ 2010000281	MZ677101
*Homatulaguanheensis*	KIZ 2005014512	MZ677105
*Homatulaguanheensis*	KIZ 2005014513	MZ677104
*Homatulalongidorsalis*	KIZ 2008005909	MZ677121
*Homatulalongidorsalis*	KIZ 2008005910	MZ677118
*Homatulavariegata*	KIZ 2009002770	MZ677110
*Homatulavariegata*	KIZ 2009002724	MZ677115
*Homutularobusta*	KIZ 2009000146	MZ677106
*Homatularobusta*	KIZ 2009000144	MZ677107
*Homatulacoccinocola*	KIZ 2012001867	MF953210
*Homatulacoccinocola*	KIZ 2012001868	MF953211
*Homatulachange*	KIZ 2015005116	MZ677109
*Homatulachange*	KIZ 2015005117	MZ677108
*Homatulacryptoclathrata*	KIZ 2005012639	MZ677116
*Homatulacryptoclathrata*	KIZ 2005012637	MZ677117
*Homatulapycnolepis*	KIZ 2009003860	MZ677111
*Homatulapycnolepis*	KIZ 20050423002	MZ677114
*Homatulaanguillioides*	KIZ 2008006539	MZ677124
*Homatulaacuticephala*	KIZ 2008005994	MZ677122
*Homatulaanguillioides*	KIZ 2008006536	MZ677125
*Homatulaacuticephala*	KIZ 2008005990	MZ677123
*Homatulawuliangensis*	KIZ 2008008160	MF953221
*Homatulawuliangensis*	KIZ 2008008159	MF953220
*Homatulawenshanensis*	KIZ 2014005686	MZ677102
*Homatulawenshanensis*	KIZ 2014005687	MZ677103
*Homatuladisparizona*	KIZ 2012000626	MF953194
*Homatuladisparizona*	KIZ 2012000622	MF953190
*Botiadario*		KT781503

## ﻿Results

### ﻿Taxonomy

#### ﻿ Homatula robusta sp. nov.

Taxon classificationAnimaliaCypriniformesNemacheilidae

EEDA864C-0C47-5F78-A3EA-D3865BBDB76D

http://zoobank.org/A27C86D0-FD58-448C-9A85-6129B1A18F66

**Figure 1.**
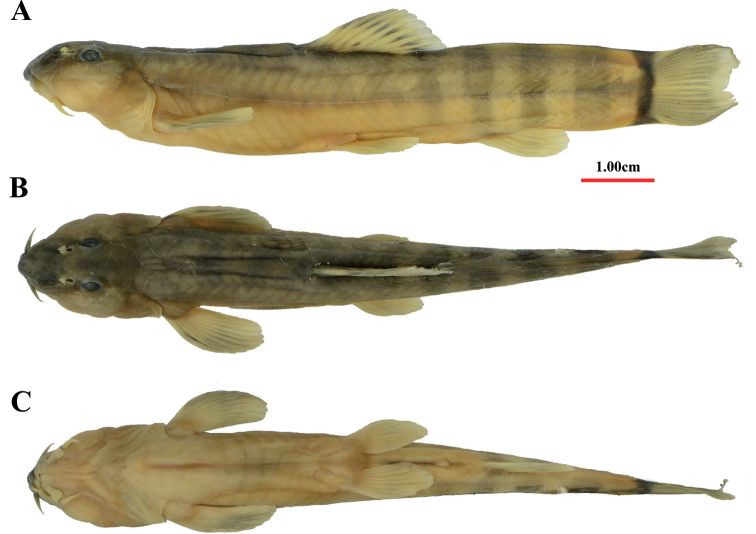
Lateral **A** dorsal **B** and ventral views **C** of *H.robusta* sp. nov., holotype, KIZ 2009000125, 83.12 mm SL.

**Figure 2.**
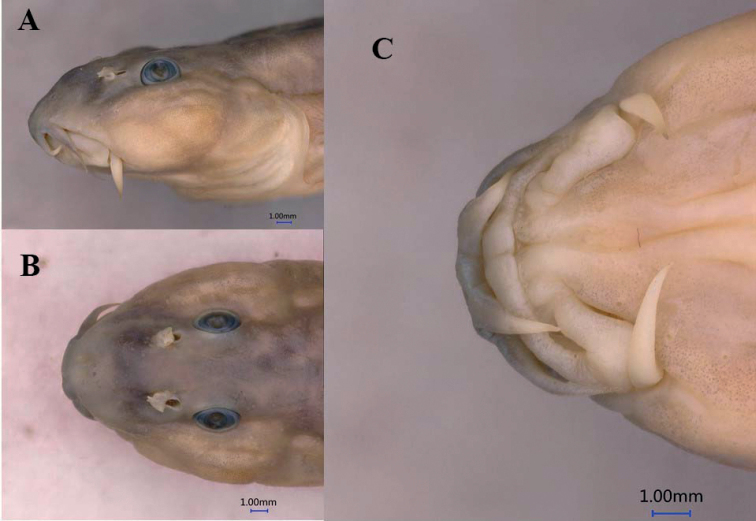
*Homatularobusta* sp. nov., paratype, KIZ 2009000122, 81.32 mm SL, head in lateral **A** dorsal **B** and ventral **C** views.

**Figure 3.**
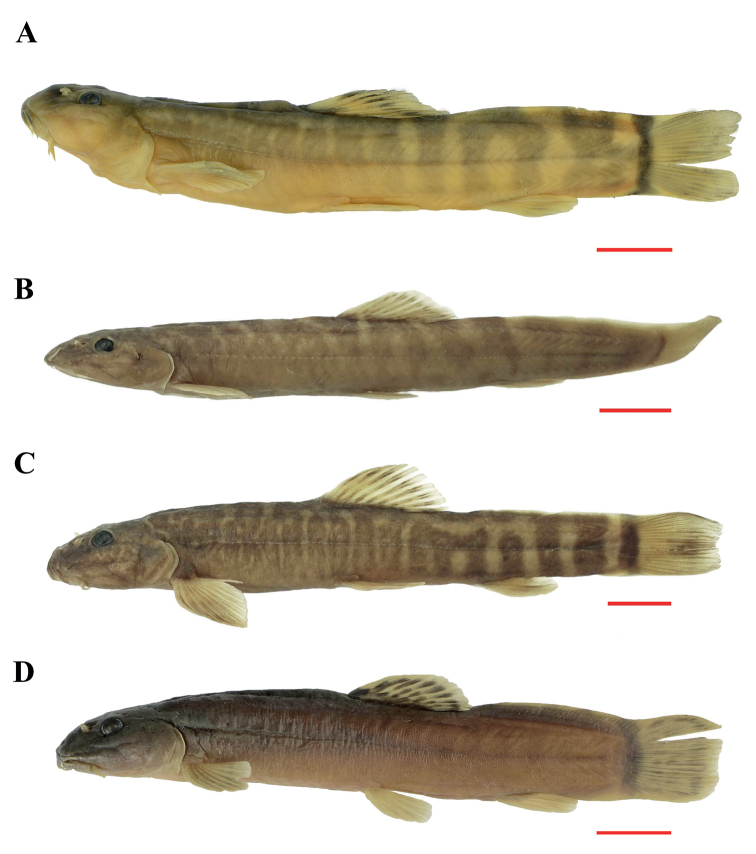
Lateral view of *H.robusta* sp. nov. KIZ 2009000122 paratype **A***H.longidorsalis*KIZ 1987003989 **B***H.nanpanjiangensis*KIZ 1994000023 **C***H.potanini*KIZ 2010000266 **D**.

**Figure 4.**
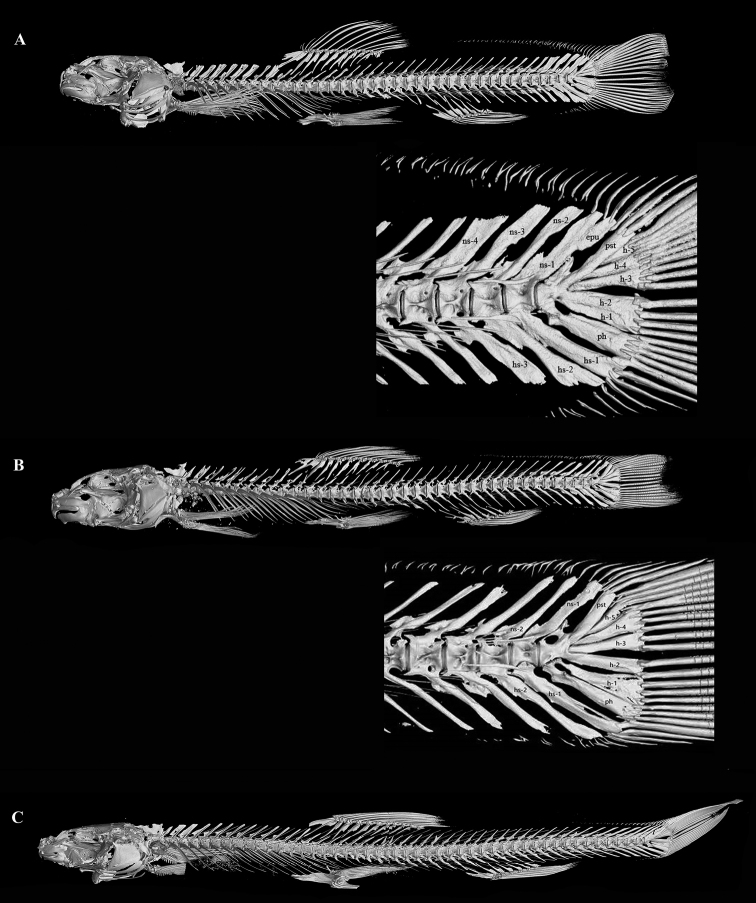
Lateral CT scans of: **A***H.robusta* sp. nov., KIZ 2009000125, lateral view and structure of caudal skeletons **B***H.nanpanjiangensis*, KIZ 1994000029, lateral view and structure of caudal skeletons **C***H.wenshanensis*, KIZ 2014005686, lateral view. Abbreviations: ph—partypurale, ns—neural spine, hs—haemal spine, h—hypural, epu—epurale, pst—pleurostyle.

**Figure 5.**
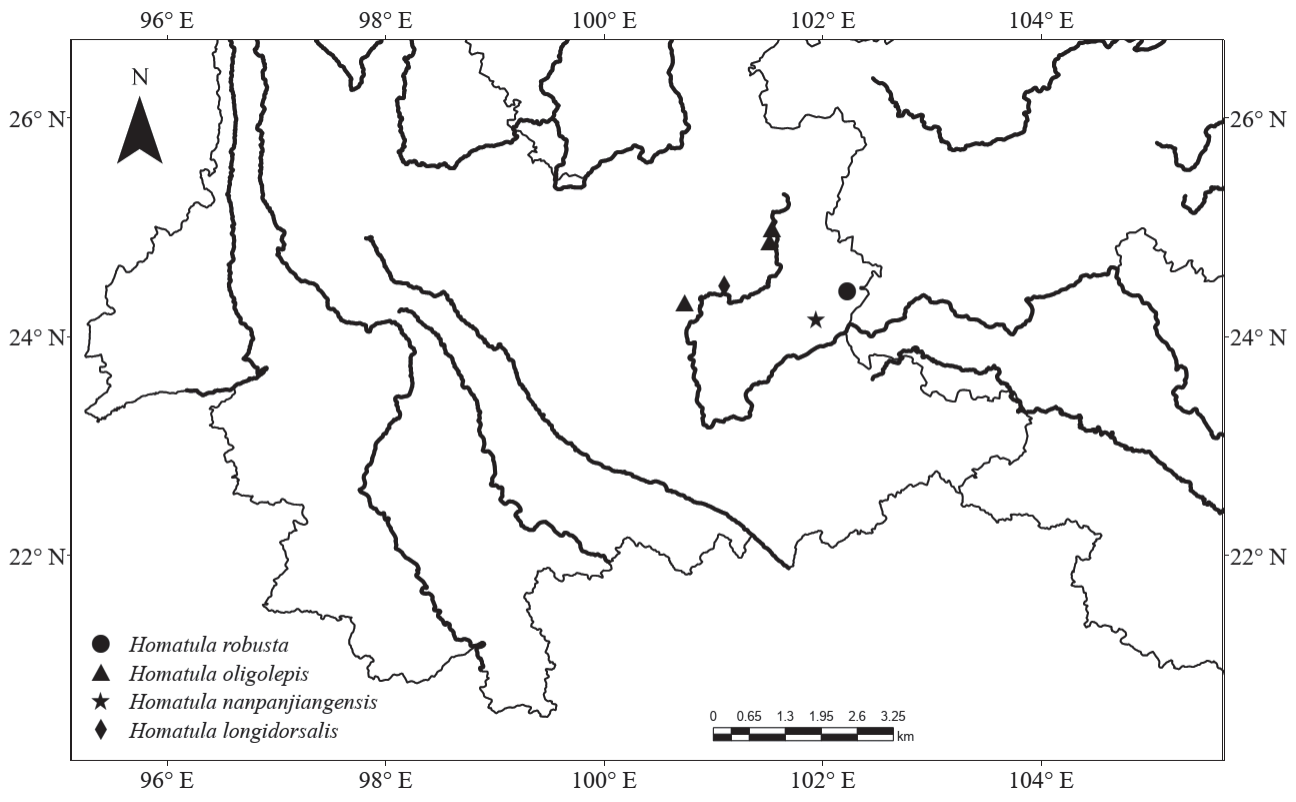
Distribution of *Homatula* from the Nanpanjiang River.

Figs 1 , 2 , 3 , 4 , 5 , Tables 2 , 3

##### Material.

***Holotype*.**KIZ 2009000125, 83.12 mm SL; collected by Wansheng Jiang and Weiying Wang on 14 March 2009 at Changdi village, Luoping County, Qujing City, Yunnan Province, China; Nanpanjiang River, upper Pearl River (25°02'N, 104°30'E; ca 1210 m). ***Paratypes*.**KIZ 2009000122, 144, 146, 3 ex. 61.16–81.32 mm SL, same data as for holotype.

##### Diagnosis.

The new species can be distinguished from all other species of *Homatula* by having the following combination of characters: naked and robust body with well-developed crests (caudal peduncle depth as a percentage of its length: 70.5–78.5%), lateral line complete, median notch on lower jaw, median gap on lower lip, three pairs of short barbels, with maxillary barbels extending posteriorly to the anterior edge of eyes, branched dorsal-fin rays 8½, emarginated caudal fin, and vertebrae 37–39.

##### Description.

Anterior body cylindrical, posterior body laterally compressed; robust, depth 5.8–6.3 times in length. Caudal peduncle stout, depth 1.27–1.42 times in its length. Crests on dorsal and ventral midlines present and supported by rudimentary procurrent caudal-fin rays; dorsal crest starting immediately posterior of dorsal-fin base, ventral crest starting immediately posterior of anal-fin base.

Snout blunt in lateral view, cheeks inflated. Eyes elliptical horizontally, dorsolaterally positioned. Mouth inferior, slightly arched. Anterior nostril in flap, next to posterior nostril. Lips moderately thick, upper lip smooth, slightly notched medially, lower lip with shallow furrows and median gap. Processus dentiformis on upper jaw present with circular arc edge; lower jaw spoon-like with a median notch. Three pairs of barbels, maxillary barbel reaching anterior margin of eye, outer rostral barbel reaching inner corner of mouth and inner rostral barbel not.

Dorsal-fin rays iv, 8½, distal margin slightly convex. Pectoral-fin rays 11, reaching about halfway from insertion of pectoral fin to insertion of pelvic fin. Pelvic-fin rays 6–8, reaching close to anus, inserted opposite of the first branched dorsal-fin ray. Anus located 1.53–2.17 times eye diameter in front of anal-fin origin. Anal-fin rays iii, 5½. Caudal-fin rays 9+8, distal margin of caudal fin emarginated with upper and lower lobes almost equal in length. Moderate axillary pelvic lobe with free tip.

**Table 2.** Measurements of four type specimens of *Homatularobusta* sp. nov.

Measurements (mm)	*H.robusta*			
	2009000125	2009000122	2009000144	2009000146
SL	83.12	81.32	73.70	61.16
Head length	18.76	18.28	16.58	13.28
Predorsal length	38.78	37.34	35.16	29.06
Preventral length	41.80	40.94	37.06	29.98
Preanal length	60.42	60.46	54.52	45.60
Preanus length	55.92	56.00	50.16	41.96
Body depth	14.40	13.82	11.72	10.28
Caudal peduncle length (CPL)	16.56	14.78	13.32	9.96
Caudal peduncle depth (CPD)	11.68	11.60	10.20	7.04
Body width	9.36	9.38	8.66	7.06
Dorsal-fin length	11.28	11.48	10.24	7.12
Anal-fin length	12.32	11.94	11.58	9.14
Pelvic-fin length	12.12	12.28	11.22	8.72
Pectoral-fin length	14.82	14.76	13.50	11.12
Head depth at neck	11.84	11.72	10.70	8.56
Snout length	8.64	8.48	7.48	6.10
Head width at eye	13.12	13.34	12.40	9.46
Max head width	14.08	13.90	12.88	10.78
Interorbital width	4.30	4.44	4.24	3.74
eye diameter	2.62	2.34	2.28	2.10

Body scaleless, or sparse scales scattered along lateral line after posterior end of anal-fiin base, embedded beneath skin. Lateral line completed with 85–89 pores. Supraorbital pores 7, postorbital pores 3, sub- and preorbital pores 12, preoperculo-mandibular pores 10, supratemporal pores 3.

Vertebrae (three specimens), 4+37–39; four hypural elements with h-1 & h-2 fused, epurale present, last four neural spines (ns-1 to ns-4) and last three haemal spines (hs-1 to hs-3) on the caudal vertebrae are significantly enlarged. U-shaped stomach; intestine almost straight, with small bend next to stomach posterior. Longest recorded length is 83.1 mm SL, 95.7 mm total length (KIZ 2009000125, holotype).

**Table 3.** Morphometrics of *Homatularobusta* sp. nov. and *Homatulananpanjiangensis*. SD, standard deviation.

	*H.robusta*					*H.nanpanjiangensis*				
Measurements	N	Min	Max	Mean	SD	N	Min	Max	Mean	SD
SL (mm)	4	61.1	83.12	74.83	8.65	19	63.82	88.74	78.37	7.56
**As percent of SL**										
Head length	4	13.28	18.76	16.73	2.15	19	15.46	20.36	18.63	1.53
Predorsal length	4	45.92	47.71	46.95	0.72	19	45.62	50.47	47.98	1.37
Preventral length	4	49.02	50.34	49.98	0.56	19	49.58	54.03	51.14	1.14
Preanal length	4	72.69	74.56	73.89	0.73	19	71.25	76.94	74.37	1.47
Preanus length	4	67.28	68.86	68.20	0.61	19	66.55	70.46	69.03	1.01
Body depth	4	15.9	17.32	16.76	0.53	19	12.64	15.30	13.77	0.73
Caudal peduncle length (CPL)	4	16.29	19.92	18.11	1.29	19	16.49	20.92	18.64	1.06
Caudal peduncle depth (CPD)	4	11.51	14.26	13.42	1.11	19	9.45	12.06	10.83	0.66
Body width	4	11.26	11.75	11.52	0.17	19	9.24	13.65	10.73	1.10
Dorsal-fin length	4	11.64	14.12	13.31	0.98	7	12.00	15.83	14.21	1.19
Anal-fin length	4	14.68	15.71	15.04	0.40	6	14.47	16.99	15.56	0.82
Pelvic-fin length	4	14.26	15.22	14.79	0.39	6	13.47	15.67	14.40	0.68
Pectoral-fin length	4	17.83	18.32	18.12	0.18	6	15.35	19.30	17.49	1.16
**As percent of head length**										
Head depth at neck	4	63.11	64.54	64.06	0.57	19	49.88	61.19	55.01	3.79
Snout length	4	45.11	46.39	45.87	0.47	19	40.92	47.69	44.16	2.28
Head width at eye	4	69.94	74.79	72.23	1.83	19	49.63	72.79	59.56	6.77
Max head width	4	75.05	81.17	77.49	2.33	19	63.22	77.27	69.29	3.72
Interorbital width	4	22.92	28.16	25.24	1.93	19	22.21	26.67	24.29	1.17
Eye diameter	4	12.8	15.81	14.08	1.09	19	13.79	17.94	15.95	1.05
**CPD/CPL (%)**	4	70.53	78.48	74.07	3.53	19	49.50	65.66	58.22	4.22

##### Coloration of preserved specimens.

Body light brown with vertical brown bars in formalin-fixed specimens. Bars on predorsal body usually blurred and indistinct, or countable and separated by extraordinary narrow interspaces just in KIZ 2009000122. Bars and interspaces getting wider towards caudal-fin base and approximately equal width on posterior body. Usually, bars regularly shaped and jointed on dorsal midline from opposite sides, or two bars met on upper body and last bar diffused or formed by two combined bars (KIZ 2009000122). Dark black bar on caudal-fin base, reaching dorsal and ventral extremities. All fin rays pale brown and covered by melanophores, series of stripes halfway up each dorsal-fin ray. Color in alcohol-fixed specimens is paler than those in formalin-fixed specimens.

##### Sexual dimorphism.

No sexual dimorphism was observed.

##### Etymology.

Robusta is a Latin word meaning ‘strong’, in reference to the stout body and caudal peduncle. The Chinese common name is suggested as 粗壮荷马条鳅.

##### Distribution.

Only known from the type locality, Changdi village, Luoping County, Qujing City, Yunnan Province, China (Fig. 5).

### ﻿Phylogenetic characterization and relationships

**Figure 6.**
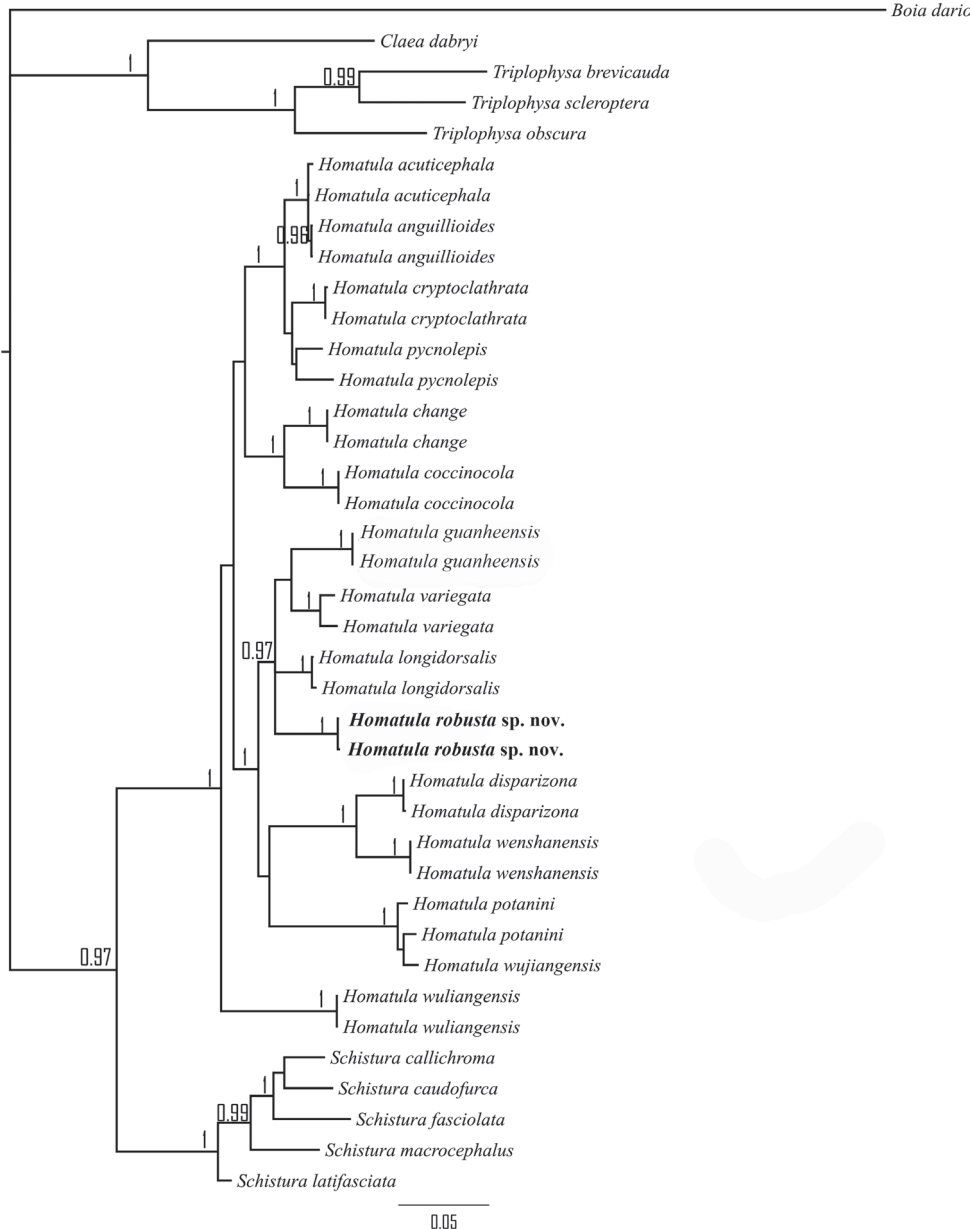
COI MrBayes phylogenetic reconstruction of *Homatula*. BI posterior probabilities of more than 0.95 are plotted on the branches.

The COI molecular dataset included 39 terminal taxa representing 25 species, 15 of which belonged to *Homatula* (Table 1). The COI gene was 1116 bp in length with 313 informative sites, 68 singleton sites, and 717 constant sites. The Bayesian inference (BI) phylogenetic analysis recovered the monophyly of *H.robusta*, *H.wenshanensis* and most species of *Homatula* (Fig. 6). The K2P distance between *H.robusta* and its closest species on the tree, *H.longidorsalis*, is 5.3%. *Homatulawenshanensis* was the sister group of *H.disparizona*. *Homatulapotanini* and *H.wujiangensis* (Ding & Deng, 1990) were clustered together, with a K2P distance of 1.5%, and *H.acuticephala* and *H.anguillioides* were clustered together, with a K2P distance of 0.2%.

## ﻿Discussion

*Homatularobusta* sp. nov. can be distinguished from its congeners except *H.disparizona*, *H.nanpanjiangensis*, *H.wujiangensis*, *H.oligolepis*, *H.dotui* and *H.wenshanensis* by body scaleless, or sparse scales scattered along the lateral line after anal-fin base (vs. anterior body scaleless or with rudimentary scales in *H.berezowskii*, *H.guanheensis*, *H.laxiclathra*, *H.longidorsalis*, *H.potanini*, and *H.variegata*; whole body scaled besides the head in *H.acuticephala*, *H.anguillioides*, *H.anteridorsalis*, *H.change*, *H.coccinocola*, *H.cryptoclathrata*, *H.nigra*, *H.pycnolepis*, *H.wuliangensis*). The new species can be distinguished from *H.dotui* and *H.wujiangensis* by the complete lateral line (vs. incomplete), presence of brown bars on the body (vs. absence in *H.dotui*), 37–39 vertebrae (vs. 31 in *H.dotui*), normally developed eye, 12.8–15.8% of HL (vs. rudimentary, 4–6% in *H.dotui*), caudal-fin rays 9+8 (vs. 8+7 in *H.dotui*), dorsal crest reaching forward beyond the origin of anal-fin base (vs. not reaching the posterior point of anal-fin base in *H.wujiangensis*) and from *H.oligolepis* by the regular bars on the side of the body (vs. vermiform markings on the head and body), 8 ½ branched dorsal-fin rays (vs. 9 ½), vertebrae 37–39 (vs. 39–41). It can be distinguished from *H.disparizona* and *H.wenshanensis* by the stronger body with BD 15.9–17.3% of SL (vs. 12.1–15.4% in *H.disparizona*, 12.1–14.8% in *H.wenshanensis*), vertebrae 37–39 (vs. 39–40 in *H.disparizona*, 47–48 in *H.wenshanensis*), stout caudal peduncle with CPD 70.5–78.5% of Caudal peduncle length (CPL) (vs. 47–62% in *H.disparizona*, 27.3–35% in *H.wenshanensis*), and the median notch on the lower jaw present (vs. absent), caudal fin slightly emarginated (vs. forked in *H.wenshanensis*). *Homatularobusta* can be distinguished from its most similar species, *H.nanpanjiangensis*, on external morphology by the stouter caudal peduncle with CPD 70.5–78.5% of CPL (vs. 49.5–65.7%), deeper body depth (BD) 15.9–17.3% of SL (vs. 12.6–15.3%), shorter barbel with maxillary barbel reaching the anterior margin of the eye (vs. between middle and posterior margin of eye) (Table 3, Fig. 3C), and differed from *H.nanpanjiangensis* on the structures of the caudal skeleton by having four hypural elements with h-1 and h-2 fused (vs. five, h-1 and h-2 separated), epurale present (vs. absent), last four neural spines and last three haemal spines of the caudal centra significantly enlarged (vs. slightly enlarged) (Fig. 4).

*Homatulawenshanensis* was questioned as member of the genus *Homatula* by Nguyen et al. (2021), because of its indistinct adipose crests along the dorsal and ventral midlines of caudal peduncle, a forked caudal fin and 4+47–48 vertebrae that are not shared by species of *Homatula*. The results of our skeleton scan showed that *H.wenshanensis* has the typical generic character of *Homatula* – crests on caudal peduncle supported by rudimentary procurrent rays (Fig. 4C) – and our COI-based phylogeny showed that *H.wenshanensis* formed an independent lineage sister to *H.disparizona*. Therefore, *H.wenshanensis* is confirmed as a species of the genus *Homatula*.

*Homatula* is previously believed to be restricted to China. Recently, *H.dotui*, a cave-dwelling species, was reported from central Vietnam (Nguyen et al. 2021). *Homatuladotui* is between *Schistura* and *Homatula* as an independent lineage in the phylogenetic tree built by the cytb gene (Nguyen et al. 2021), which indicates that this cavefish species could belong to an undescribed genus. As stated by Nguyen et al. (2021: 8), a further study should be addressed to confirm the placement of *H.dotui*.

Three species of *Homatula* have been previously reported from the Nanpanjiang River: *H.oligolepis* and *H.longidorsalis* are distributed in the upper Nanpanjiang River; *H.nanpanjiangensis* is distributed in the middle Nanpanjiang River. They possess an elongate body of medium to large size, scaleless (*H.oligolepis* and *H.nanpanjiangensis*) or at least scaleless on the predorsal body (*H.longidorsalis*), 9 ½ branched dorsal-fin rays (*H.oligolepis* and *H.longidorsalis*) or 8 ½ (a few 9 ½ in *H.nanpanjiangensis*), regular vertical bars on each side of body, and bars in front of dorsal-fin base conspicuously thinner than those behind (*H.longidorsalis* and *H.nanpanjiangensis*) or vermiform markings on body and dorsal head (*H.oligolepis*). Here, *H.robusta* sp. nov. is reported from the middle Nanpanjiang River with a stout body. For better identification, a key to species distributed in the Nanpanjiang River is provided.

### ﻿Key to species of *Homatula* in the Nanpanjiang River

**Table:** 

1	Body scaleless or with rudimentary scales present at caudal peduncle	**2**
–	Scales clearly present, covering posterior of body at least, anterior nostril in short tube, 9 ½ branched dorsal-fin rays	***H.longidorsalis***
2	Medium-sized body with regular bars on body, interspaces thinner than bars on predorsal body, SL up to 88.7 mm	**3**
–	Large-sized body with vermiform markings on body and head, SL up to 170.7 mm	***H.oligolepis***
3	Well-developed crests with CPD 70.5–78.5% of CPL, maxillary barbel reaching anterior margin of eye, no more than 13 bars, four hypural elements, epural present, last four neural spines and last three haemal spines on caudal vertebrae significantly enlarged	***H.robusta* sp. nov.**
–	Medium crests with CPD 49.5%–65.7% of CPL, maxillary barbel reaching between middle and posterior margin of eye, ~16 bars on average, five hypural elements, epurale absent, neural and haemal spines on caudal vertebrae slightly enlarged	***H.nanpanjiangensis***

## Supplementary Material

XML Treatment for Homatula robusta
